# Developmental and neurochemical features of cholinergic neurons in the murine cerebral cortex

**DOI:** 10.1186/1471-2202-10-18

**Published:** 2009-03-09

**Authors:** Silvia Consonni, Silvia Leone, Andrea Becchetti, Alida Amadeo

**Affiliations:** 1 Department of Biotechnology and Biosciences, Piazza della Scienza 2, University of Milano, Bicocca, 20126 Milano, Italy; 2 Department of Biomolecular Sciences and Biotechnology, Via Celoria 26, University of Milano, 20133 Milano, Italy

## Abstract

**Background:**

The existence and role of intrinsic cholinergic cells in the cerebral cortex is controversial, because of their variable localization and morphology in different mammalian species. We have applied choline acetyltransferase (ChAT) immunocytochemistry to study the distribution of cholinergic neurons in the murine cerebral cortex, in the adult and during postnatal development. For more precise neurochemical identification of these neurons, the possible colocalization of ChAT with different markers of cortical neuronal populations has been analyzed by confocal microscopy. This method was also used to verify the relationship between cholinergic cells and cortical microvessels.

**Results:**

ChAT positive cells appeared at the end of the first postnatal week. Their density dramatically increased at the beginning of the second postnatal week, during which it remained higher than in perinatal and adult stages. In the adult neocortex, cholinergic neurons were particularly expressed in the somatosensory area, although their density was also significant in visual and auditory areas. ChAT positive cells tended to be scarce in other regions. They were mainly localized in the supragranular layers and displayed a fusiform/bipolar morphology.

The colocalization of ChAT with pyramidal neuron markers was negligible. On the other hand, more than half of the cholinergic neurons contained calretinin, but none of them expressed parvalbumin or calbindin. However, only a fraction of the ChAT positive cells during development and very few in adulthood turned out to be GABAergic, as judged from expression of GABA and its biosynthetic enzymes GAD67/65. Consistently, ChAT showed no localization with interneurons expressing green fluorescent protein under control of the GAD67 promoter in the adult neocortex. Finally, the cortical cholinergic cells often showed close association with the microvessel walls, as identified with the gliovascular marker aquaporin 4, supporting previous hypotheses on the role of cholinergic cells in modulating the cortical microcirculation.

**Conclusion:**

Our results show that the development of the intracortical cholinergic system accompanies the cortical rearrangements during the second postnatal week, a crucial stage for the establishment of cortical cytoarchitecture and for synaptogenesis. Although intrinsic ChAT positive cells usually expressed calretinin, they displayed a variable GABAergic phenotype depending on marker and on cortical developmental stage.

## Background

Cholinergic transmission in the mammalian cerebral cortex is thought to play an important role in controlling the transitions towards more vigilant brain states, with implications for learning, memory and neuropathology [1]. Most of the cholinergic innervation comes from fibres originating from basal forebrain nuclei. However, early immunocytochemical work suggested the existence of intracortical cholinergic neurons, in the rat [2-5]. Subsequently, the variability of the localization and morphology of cholinergic cells in different mammalian species has given rise to controversy about their morphological and neurochemical nature [6]. Immunocytochemical localization of choline acetyltransferase (ChAT) labels mainly, but not exclusively, neurons in the II and III layers in the rat [3,7], rabbit [8], cat [9], fetal *Macaca mulatta *[10], and different murine strains [11,12]. Cholinergic cells with pyramidal shape have also been observed in the III and V layers of the human cerebral cortex [13,14].

The function of these cells is unclear. A recent morphofunctional study addressed the physiological role and the neurochemical features of neocortical cholinergic neurons, in the mouse [15]. In particular, electrophysiological results show that ChAT positive (ChAT+) cells are innervated by both interneurons and pyramidal cells, whereas their synaptic output on these cells is negligible. However, prolonged activation of cholinergic neurons increases the frequency of the spontaneous excitatory postsynaptic currents recorded on adjacent pyramidal neurons, by an indirect effect mediated by presynaptic nicotinic receptors. These results indicate a role of ChAT+ cells in local control of cortical microcircuits.

With regard to the neurochemical nature of ChAT+ cells, earlier morphological and neurochemical data in the rat suggested they were mainly GABAergic [16,17], leading to the hypothesis that acetylcholine release could modulate the local inhibitory circuits. In the mouse, however, von Engelhardt et al. [15] showed that the expression of the mRNA for the biosynthetic enzyme for GABA (GAD67) is negligible in ChAT+ cells, although a fraction of them does express other typical interneuronal markers such as the vasoactive intestinal peptide [3,17,18] and calretinin [15,19]. The vasoactive intestinal peptide-expressing cortical interneurons control microvascular dilatation in the rat [20]. Although the main source of perivascular cholinergic innervation is the basal forebrain [21], experiments carried out with lesion approaches confirm that a fraction of the cholinergic cells could contribute to the local regulation of the cortical microvascular bed [22,23]. Nevertheless, in contrast to the basal forebrain neurons that innervate large areas of cerebral cortex, cortical cholinergic neurons seem to be ideally suited for a restricted modulatory role on small cortical columnar units [15].

Considering that cholinergic transmission plays important roles in shaping neuronal circuits during development [24], new indications about the possible functions of intrinsic cholinergic cells should come from studying the timing of appearance of these cells and their distribution during development. To this purpose, we have studied the distribution of cholinergic neurons in the murine cerebral cortex, in adult and during postnatal development, by using ChAT immunocytochemistry. To produce a fuller neurochemical identification, the colocalization of ChAT with markers for interneurons and pyramidal neurons was tested by confocal microscopy. Pyramidal neurons were identified with SMI32 antibody against the non-phosphorylated epitope of the heavy subunit of mammalian neurofilaments. Interneurons were investigated by labelling GABA, GAD67, GAD65 (two isoforms of the glutamic acid decarboxylase, biosynthetic enzyme for GABA) and the calcium binding proteins (CaBPs) parvalbumin, calbindin and calretinin. As an alternative way to identify GABAergic interneurons, we have used transgenic mice expressing green fluorescent protein (GFP) under the control of the GAD67 gene promoter [25]. Finally, the relationship of the cortical ChAT+ neurons with microvessels was investigated by means of aquaporin 4, which is the predominant water channel in the brain and is mainly expressed on the astrocytic end feet that contact the cortical microvessels [26].

The cortical cholinergic cell density quickly rised between postnatal day (P)4 and P8, was maintained for about two weeks and then slightly declined in the adult. These cells were mostly localized in the sensory cortical areas, especially in the upper layers. Although they usually expressed calretinin, they displayed a variable GABAergic phenotype depending on marker and on cortical developmental stage.

## Results

### Adult neocortex

**Figure 1:**
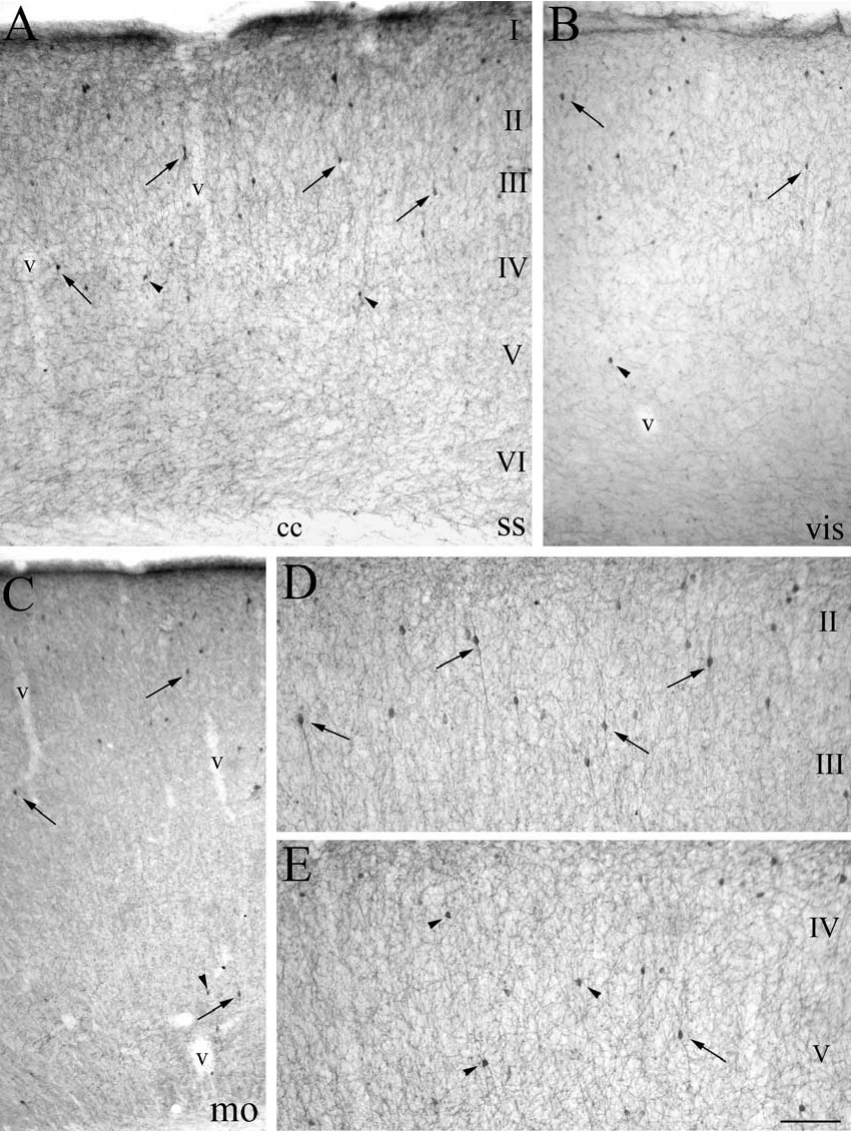
**ChAT+ neurons in the adult murine cerebral cortex**. **A**: laminar distribution of cholinergic cells in the somatosensory cortex (ss), where they appear to be widely distributed in the different layers, but more concentrated in II-III and lying sometimes in proximity of vessel (v) wall. ChAT+ cells display fusiform bipolar (arrows) or round multipolar (arrowheads) shapes throughout the cortical mantle (**B, C, D, E**). **B, C**: in the visual cortex (vis, **B**) cholinergic cells show the same distribution and density observed in the somatosensory (ss) one, whereas they are fewer and more scattered in the motor cortex (mo, **C**). **D, E**: details of supragranular layers (II-III, **D**) and deep layers (IV-V, **E**), showing the prevalence of numerous bipolar ChAT+ neurons in the former (**D**) and only a few immunopositive cells, both bipolar (arrows) and multipolar (arrowheads), in the latter (**E**). Scale bar: 112 μm in **A**; 135 μm in **B **and **C**; 87 μm in **D **and **E**.

**Figure 2:**
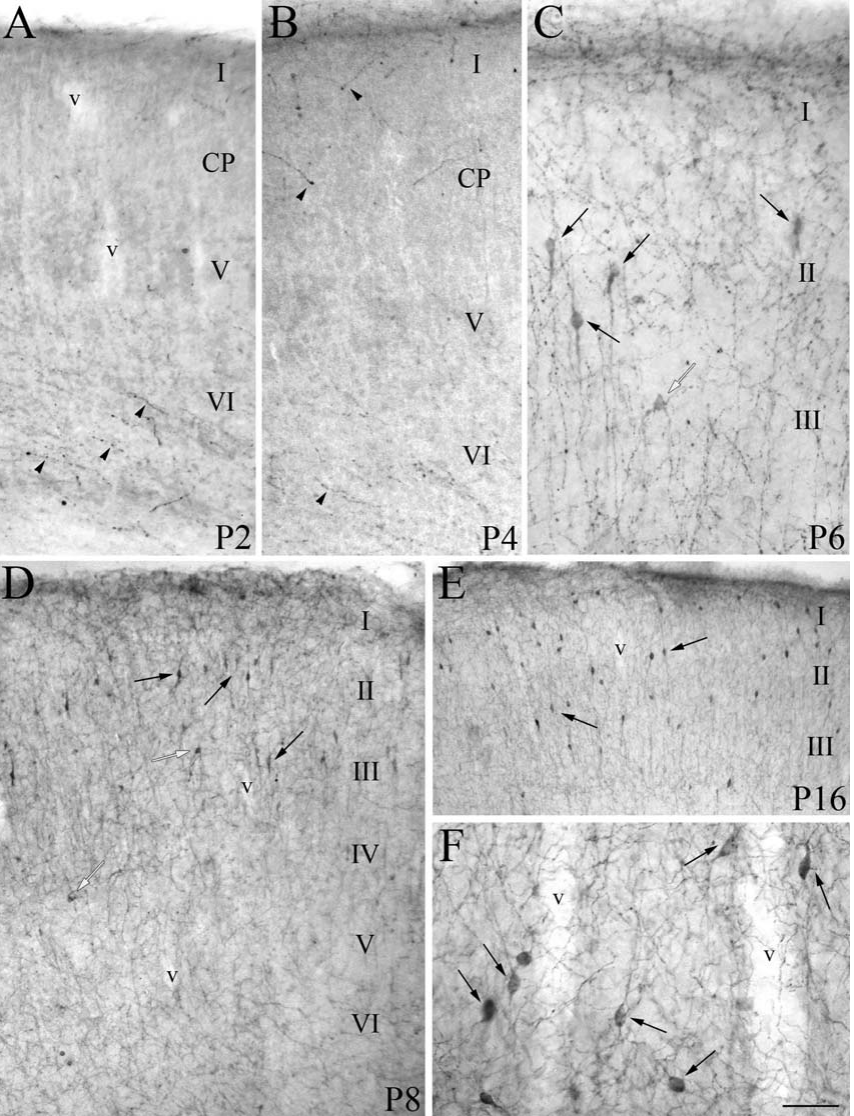
**ChAT+ neuron distribution in the developing somatosensory cerebral cortex of mouse**. **A, B**: at P2 (**A**) and P4 (**B**) very few thin varicose ChAT+ fibers (arrowheads) are observed in the process of invading the developing cortex. At P4, in particular, a few cholinergic growth cones (arrowheads) can be discerned within the cortical plate (CP). **C, D**: from P6 (**C**) to P8 (**D**) an increasing number of fusiform (arrows) and rare multipolar (white arrows) ChAT+ cells populate the supragranular layers of developing cortex. **E, F**: at P16 the cholinergic neuronal population is mainly localized in the superficial portion of the cortical mantle (**E**) and some ChAT+ cells (arrows in **F**) lie near the vessel (v) walls. Scale bar: 66 μm in **A**; 55 μm in **B**; 45 μm in **C**; 143 μm in **D**; 192 μm in **E**; 100 μm in **F**.

**Figure 3:**
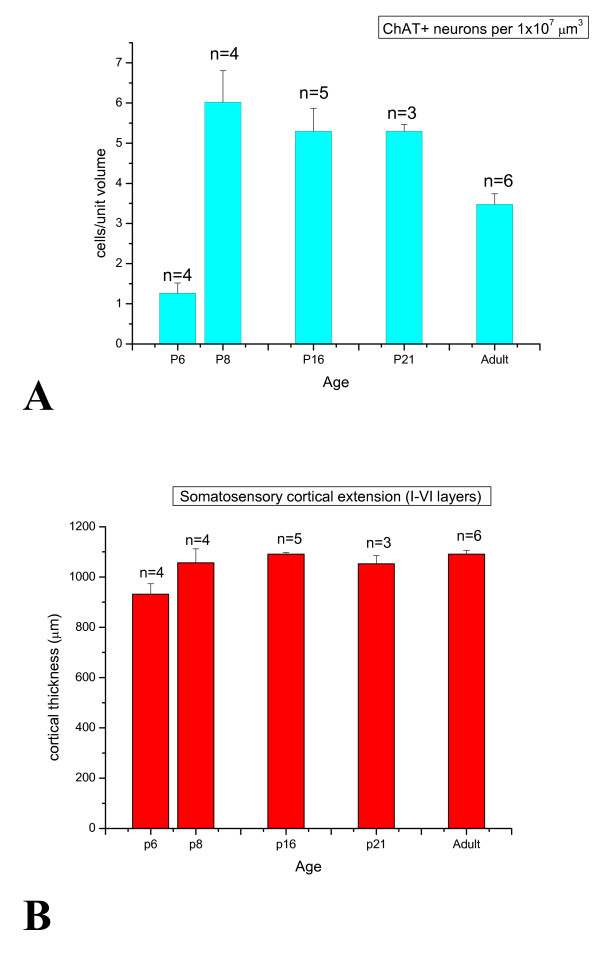
**Time course of ChAT+ cell density (A) and the corresponding cortical thickness (B), during development**. **A**: bars indicate average ChAT+ cell values per unit cortical volume, at the indicated stage. A sharp increase of cholinergic neurons was observed between P6 and P8, followed by a slow progressive decrease during late postnatal development and early adulthood. **B**: bars give the average cortical thickness at different developing stages. This parameter remained almost constant during the time span that we have studied, which shows that the time course of the cholinergic population illustrated in panel **A **is genuine.

**Figure 4:**
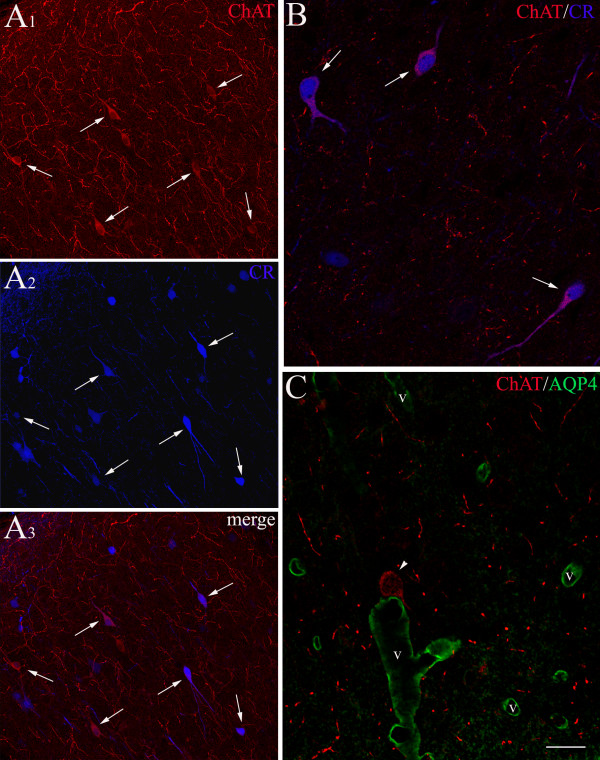
**Confocal microscopy of double immunofluorescent staining for ChAT and calretinin (CR) or aquaporin 4 (AQP4) in developing somatosensory cortex**. **A**_**1–3**_, **B**: at P12, the cortical sections immunolabelled for ChAT (red) and CR (blue) show that, after acquisition of serial optical sections, most cholinergic neurons (**A**_**1**_) display also CR immunoreactivity (**A**_**2**_). This is shown in the cells indicated by the white arrows both in **A_1-2 _**and in the combination of the two signals (merge, **A**_**3**_). **B **shows a high magnification detail of ChAT+/CR+ cortical neurons (white arrows). **C**: at P16, double immunolabelling for ChAT (red) and AQP4 (green) points out a ChAT+ cell (white arrowhead) lying on the wall of one of the numerous vessels (v) identified by the gliovascular marker AQP4. Note also the presence of several cholinergic fibers (red) in the cortical neuropile. Scale bar: 54 μm in **A**_**1–3**_; 22 μm in **B**; 17 μm in **C**.

ChAT immunolocalization showed a significant population of neurons among a dense and intricate network of varicose cholinergic fibers distributed throughout the cortical layers (Figure 1). ChAT+ cells had mostly fusiform/bipolar shape in the supragranular layers (Figure 1D), where they were more concentrated. Scattered multipolar ChAT+ neurons were also observed, especially in the deepest layers (Figure 1E). Irrespective of developmental stage, ChAT+ neurons were always observed within the II and III cortical layers, which display the highest density of cholinergic fibers and contain radial vessels entering the cortical parenchyma (Figure 1A and 1C and Figure 2F). ChAT+ neurons were particularly dense in the somatosensory cerebral cortex (Figure 1A). In this region, the average cholinergic cell concentration turned out to be 3.5 ChAT+ cells/10^7 ^μm^3 ^of cortical tissue (Figure 3). The visual (Figure 1B) and auditory regions also presented a prominent cholinergic neuronal population, whereas the incidence of ChAT+ cells was lower in the motor cerebral cortex (Figure 1C) and in several limbic areas, such as retrosplenial, cingulated, perirhinal and piriform cortices. In these, ChAT+ neurons were concentrated in the II layer (not shown). Both in adulthood and during postnatal development (see below) a fraction of these cells were found nearby the cortical vessel walls (Figures 1A, 2F and 4C).

### Postnatal development

In the developing somatosensory cortex (Figure 2), cholinergic cells appeared between P4 and P6 (Figures 2A, 2B, 2C and 3A). Subsequently, their density considerably increased around the beginning of the second postnatal week (P8; Figures 2D, 2E and 3A), especially in the supragranular layers (Figure 2D), where they assumed the typical bipolar morphology. To estimate precisely the cholinergic cell density at the different stages, it is necessary to take into account the effect of dilution due to brain growth. Therefore, we have measured the thickness of layers in the somatosensory cortex in all the cortical fields that we have considered (Figure 3B). This procedure highlighted a genuine increase of ChAT+ neuronal population between P6 and P8. Around P10, ChAT+ cells started to populate the deep layers. At late postnatal stages (P16, P21; Figure 2E) they showed density values slightly lower than at P8, but still higher than those measured in the adult (Figure 3A).

### Confocal analysis

**Figure 5:**
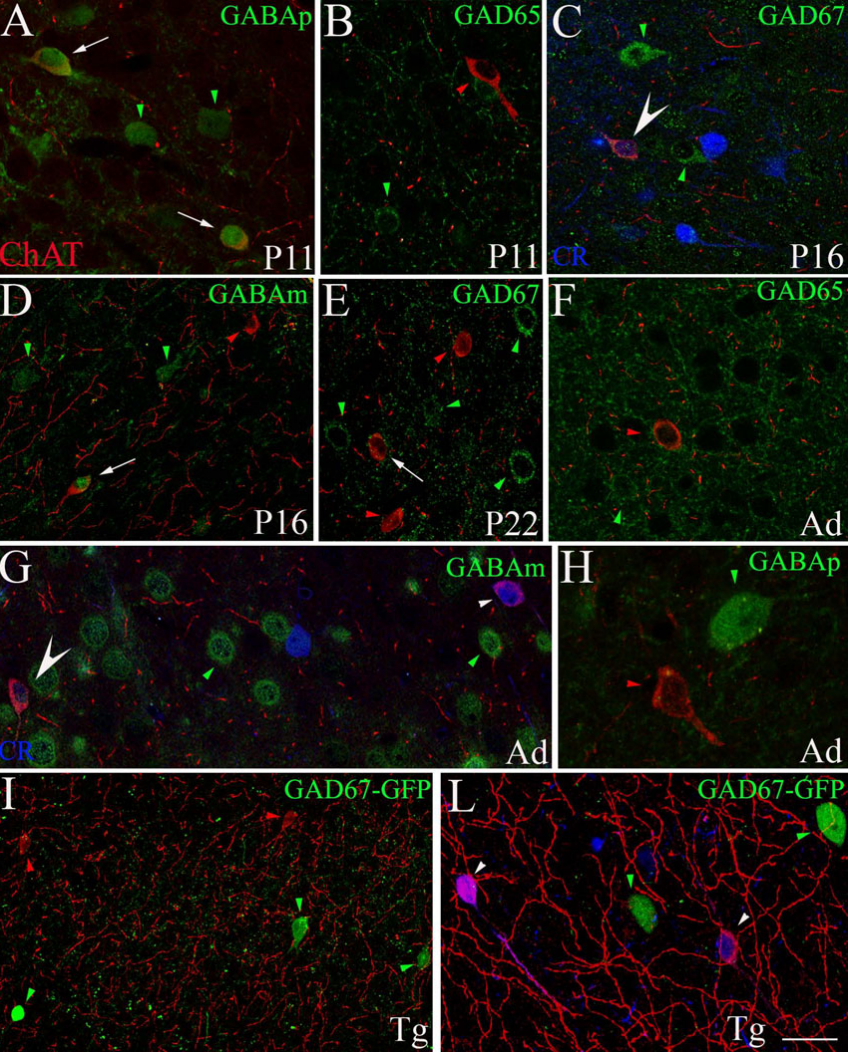
**Confocal microscopical analysis of ChAT+ neurons labelled with calretinin (CR) and different GABAergic markers**. **A, B, C, D, E**: during postnatal development at P11 (**A, B**), P16 (**C, D**) and P22 (**E**), ChAT+ cells (red) sometimes also contain (white arrows) GABAergic markers (green). GABA was revealed with either a polyclonal (GABAp in **A**) or a monoclonal (GABAm in **D**) antibody. Panels **C **and **E **show labelling of GAD67; in particular the large white arrowhead in **C **indicates a neuron that presented triple immunoreaction for ChAT/GAD67/CR (blue). No double labelling was found using GAD65 as GABAergic marker (**B**). In the adult (Ad), ChAT+ cells (red) only showed occasional immunoreactivity for GABAergic markers (green), as shown in **F **(GAD65), **G **(GABAm) and **H **(GABAp). In **G**, the large white arrowhead indicates again a cell labelled also for CR (blue). **I, L**: in the adult transgenic (Tg) GAD67-GFP mouse, no ChAT+ neurons (red) expressed GFP (green), but two of them contained CR (blue signal in **L**, small white arrowheads). Small red arrowheads: single ChAT+ neurons. Small green arrowheads: single GABAergic cells. Small white arrowheads: double ChAT+/CR+ neurons. Scale bar: 23 μm in **A**, **B **and **E**; 20 μm in **C**, **F **and **G**; 33 μm in **D **and **I**; 11 μm in **L**.

Double immunolabelling experiments (Figures 4 and 5) showed that, both in adulthood and during postnatal development, the somatosensory murine cerebral cortex was populated by ChAT+ cells that did not display any immunoreactivity for either SMI32, or CaBPs parvalbumin and calbindin, or GAD65 (Figure 5B and 5F). Instead, more than half of these cells resulted to be calretinin positive (Figures 4A_1–3_, 4B, 5C, 5G and 5L), although their response to GABAergic markers turned out to be rather variable (Figure 5). During development most cholinergic cells showed immunoreactivity with the polyclonal anti-GABA antibody (Figure 5A), but little reaction was obtained when using a monoclonal antibody (Figure 5D). Little signal was also observed when labelling GAD67 (Figure 5C and 5E). In the adult, the colocalization of ChAT+ signal and GABA became scarce even with the polyclonal antibody (Figure 5F, 5G and 5H). To obtain an independent test of the relation between cholinergic and GABAergic markers, we have repeated our tests in the somatosensory cortex of adult GAD67-GFP mice (a strain expressing GFP under the control of the GAD67 promoter; [25]). In agreement with the results illustrated above, the cholinergic neurons did not express GFP, thus suggesting that a good part of them were not GABAergic (Figure 5I and 5L). Combining ChAT immunolocalization with labelling for aquaporin 4 (Figure 4C), we observed that cholinergic fibers and puncta were often observed in close relationship with aquaporin 4 positive glial endfeet. Moreover, some of the cholinergic fusiform neurons were closely apposed to the walls of arterioles and capillaries.

## Discussion

### Cholinergic cell distribution in the adult neocortex

Overall, our data highlighted a cortical cholinergic cell population with features broadly comparable to those described by previous work on rodents [3,5,12,18]. The comparison with other nonhuman mammalian species shows a general similarity in the bipolar morphology of ChAT+ cells and a prevalent localization in the II and III layer, whereas the cells' distribution in neocortical regions is slightly different between species. In cats, rabbits and fetal monkeys, ChAT+ cells are fewer, compared to rodents, and appear to be distributed homogeneously also in motor and limbic areas [8-10]. In the human cortical mantle, they were mainly pyramidal and expressed in the motor cerebral cortex [13]. Although some of these differences might depend on use of different antibodies, in general they can be probably attributed to a progressive evolutionary change of the intrinsic cortical cholinergic system, because of the functional requirements of the neocortex of different mammalian species [12]. In the cat neocortex, for example, a strict connection of intrinsic cholinergic cells with neuronal activity after sensory stimulation has been demonstrated by experiments carried out with positron emission tomography [23].

Another common feature of cholinergic neurons is their frequent apposition to the walls of microarterioles and capillaries. This was confirmed by our immunofluorescence experiments on adult murine cerebral cortex, in analogy to what has been observed in the adult rat and human cortical regions [3,14,18].

### Cholinergic cells during development

In the somatosensory cortex, the expression of ChAT immunoreactivity during postnatal development showed a slow onset during the first postnatal week. We have carried out similar tests in the rat and observed a considerable number of cholinergic neurons at P4 and P6 (Amadeo, unpublished results), in agreement with a recent analysis [27], which showed an appreciable ChAT+ population in rat cerebral cortex at P4.

At the beginning of the second postnatal week, the ChAT+ cells' expression showed an upsurge. Such a timing parallels the rapid invasion of cholinergic fibers known to take place in the rat during the first two postnatal weeks [27] and the quick rise in the expression of α3 and α7 nicotinic receptor subunits, in the forebrain [24]. Therefore, we speculate that local acetylcholine release by cortical cholinergic cells might contribute to regulate synaptic refinement in restricted regions. This could adjust the nicotinic receptor expression [28], with possible regulatory effects on firing of the neighbouring cells [15]. Through these or other mechanisms, cholinergic cells could influence the tuning to sensory inputs of the developing cortical circuitry [24].

A frequent association between ChAT+ cells and blood vessels was also observed during the second postnatal week, which is suggestive because maximal sprouting of new cortical vessels occurs during this stage [29-31]. This phase is characterised by cerebral remodelling, accompanied by a progressive increase in neuronal activity. This process boosts local metabolic needs that need to be suited by neoangiogenesis [29]. The vascularity of cortical sensory areas is also characterised by marked interlaminar differences [32]. The highest vessel density is observed in the superficial layers of the somatosensory barrel cortex [33], where we observe most of the cholinergic neurons. Considering that the second postnatal week is the critical period for major developmental events, such as synaptogenesis, spine formation and also gliogenesis [32,34], we hypothesise that cortical ChAT+ cells could also regulate the vascular tone in this critical stage.

### Neurochemical features of cortical cholinergic neurons

Cortical ChAT+ cells were originally hypothesised to be a subpopulation of the GABAergic cortical interneurons [16,17], mainly because of their morphology (mostly bipolar, rarely multipolar). Our results with SMI32 antibody support the idea that these cells are not pyramidal neurons. However, a recent study suggests that in young adult mice most of the cholinergic cells are not GABAergic either, because they do not show appreciable expression of GAD67 mRNA [15]. These cells may nonetheless derive from cell populations that are GABAergic in early stages [35,36]. Our immunocytochemical experiments indicate that at least a fraction of ChAT+ neurons, more frequently in adulthood, display neither GABA nor GAD67/65 immunoreactivity. Consistent results were obtained by applying an independent method, i.e. studying ChAT expression in GAD67+ cells identified by GFP expression, in transgenic murine strains. In conclusion ChAT+ cells could represent an interneuronal subpopulation showing variable GABAergic features in different phases of cortical development.

These observations must be considered in the context of the data obtained with calretinin labelling. More than half of ChAT+ neurons turned out to express calretinin, something especially evident during the second postnatal week, when the presence of a considerable number of cholinergic cells facilitated our survey. Recent papers show that calretinin is expressed in most of adult cholinergic cortical neurons from rat and mouse [15,19]. Therefore, keeping in mind the kinetics of ChAT+ cell appearance (Figure 3A), we conclude that the time-courses of calretinin and ChAT expression in cortical cholinergic cells overlap. The timing of calretinin expression is similar in the rat [37,38], although it has not been correlated with the expression of ChAT. It is worth mentioning that the calretinin positive cortical population displays some unique developmental features, among the cortical GABAergic interneurons. First, the bipolar calretinin expressing neurons are mainly generated from the caudal part of the ganglionic eminence [36,39,40] or even from the subventricular zone [36,41], whereas most cortical GABAergic neurons originate from the medial ganglionic eminence. Second, they exhibit peculiar developmental and neurochemical properties, such as postnatal increase and variability of GABA and GAD67/65 expression [37]. It is worth noting that a late postnatal upregulation of calretinin expression in murine bipolar cortical neurons expressing GAD65 has been recently demonstrated [42]. In our hands, antibodies against GAD65 gave poor results in localizing neuronal cell bodies. However, a weaker signal of GAD65 compared to GAD67 is not surprising, if one considers that the former has been reported to target preferentially the nerve terminals, whereas the latter is more widely distributed in cells [43]. Nevertheless, it is possible that a fraction of the ChAT+ cortical neurons belong to a GABAergic subpopulation that migrates and expresses calretinin only in late postnatal development [42,44].

## Conclusion

Cholinergic neurons in the murine cerebral cortex quickly appeared at the boundary between the first and the second postnatal week, with higher density in the somatosensory areas and frequent association with microvessels. They persisted in the adult age, although with a lower density (about 50% of the peak at P8). These cells often expressed calretinin, and the time course of ChAT and calretinin appearance proceeded in parallel. The ChAT+ coexpression with GABA and GAD67 was less frequent. Basing on the pattern and timing of their appearance, we hypothesise that the cortical cholinergic cells perform a regulatory role during the cortical maturation at a sensitive stage. They could participate in the coordination of massive waves of sensory stimuli, neuronal activation, increased blood flow and progressive formation of topographical cortical sensory maps.

## Methods

### Preparation of cortical sections

We have studied thirty mice (FVB, Harlan, Italy), ranging from P2 to P40 (adult). Experiments were carried out in accordance with the guidelines established in the Principles of Laboratory Animal Care (directive 86/609/EEC) and all efforts were made to reduce the number of animals used. After inducing deep anaesthesia with chloral hydrate (4%, 2 mg/100 g i.p.), animals were sacrificed by intracardiac perfusion of 4% paraformaldehyde in phosphate buffer. For some animals (P7 and P40) a fixative solution with the addition of 0.5% glutaraldehyde was used to achieve better results with the polyclonal antibody anti-GABA (see below). Brains were removed and immersed in fixative medium overnight at 4°C. Coronal sections (50 μm thick) were cut with a Vibratome (VT1000S, Leica). At least 3–4 brain sections from different cortical regions, typically visual, auditory, somatosensory and motor, were selected for immunocytochemistry. Cytoarchitectonic controls were carried out according to the atlas of murine brain [45], on alternate sections adjacent to those processed for immunocytochemistry and stained with thionin. Sections from cerebral cortex of GFP-GAD67 transgenic mice (gift from Z. Josh Huang, Cold Spring Harbor Laboratory, NY, [25]), were also used for double immunofluorescence experiments.

### Single immunocytochemistry

Cortical cholinergic cells were localized by using a goat polyclonal anti-ChAT antibody (AB144P, Chemicon International Inc., 1:500), known to give reliable labelling in rodent cerebral cortex [12]. After aldehydes quenching with NH_4_Cl and inactivation of endogenous peroxidases with H_2_O_2_, sections were permeabilised with 0.2% Triton X-100, in 10% normal goat serum for 30 min. Subsequently, they were incubated overnight with the primary antibody, at room temperature. This procedure was followed by incubation with biotinylated anti-goat IgG (Vector Inc., diluted 1:200), for 75 min. After washing, sections were treated with the avidin-biotinylated complex (ABC kit, Vector Inc., diluted 1:100) and then with a freshly prepared solution (0.075%) of 3-3'-diaminobenzidine tetrahydrochloride (Sigma Aldrich) and 0.002% H_2_O_2_. Finally, the sections were mounted, dehydrated and layed on coverslips. The specificity of anti-ChAT antibody was checked by processing some sections in the absence of the primary antibody, or with anti-ChAT antibody previously adsorbed with rat recombinant protein (AG220, Chemicon International Inc., 4 μg/ml). In these cases, no specific staining was ever observed. Specificity for the same antibody was also tested with immunofluorescence experiments (see below). The sections processed for single immunolabelling were examined under the light microscope (Zeiss). After a qualitative evaluation of cortical cholinergic cells at the different murine developmental stages (Figures 1 and 2) at least twelve images from different fields of somatosensory cortex from P6, P8, P16, P21 and adult mice were acquired to perform by an image analysis software (Image J) a quantitative estimation of developing cortical thicknesses and of cholinergic neurons (Figure 3). The ChAT+ cells were counted by two observers independent each other to obtain a mean value of cholinergic neurons per unit of cortical volume (1 × 10^7 ^μm^3^), as reported in previous papers [5,18].

### Double immunofluorescence experiments

ChAT immunocytochemistry was coupled to treatment with different neuronal and glial markers. To label cortical interneurons, we have used monoclonal antibodies against GABA (Sigma Aldrich, 1:300), GAD65 (Chemicon, 1:300) and against calretinin (SWant), calbindin (SWant) and parvalbumin (Sigma Aldrich), diluted 1:2000. For some double immunolabellings also polyclonal anti-calretinin (1:1500, SWant), anti-GABA (1:5000, Sigma Aldrich) and anti-GAD67 (1:300, Immunological Sciences) were used. To label pyramidal neurons, we have used monoclonal SMI32 antibody (Sternberger Monoclonals), diluted 1:1000 and to reveal cortical microvessels the polyclonal anti-aquaporin 4 antibody (Sigma Aldrich), diluted 1:250.

Sections from selected developmental stages (P11, P12, P16, P22, adult) and from GAD67-GFP transgenic mice were permeabilised for 30 min with 0.2% Triton X-100, diluted in PBS containing 1% bovine albumin serum, and incubated overnight in a mixture of anti-ChAT plus one of the antibodies listed above. The sections were subsequently incubated for 2 hours in a mixture of the appropriate corresponding secondary antibodies, at room temperature. ChAT antibody was always revealed with sequential treatment with biotinylated horse anti-goat IgG (Vector Inc.) and Alexa-488 or Red X labelled streptavidin (1:200, Molecular Probes). The primary monoclonal (anti-GABA, anti-GAD65, anti-CaBPs and SMI32) antibodies were detected with indocarbocyanine Cy3-conjugated donkey anti-mouse, whereas the polyclonal (anti-calretinin, anti-GABA, anti-GAD67 and anti-aquaporin 4) antibodies were identified with indocarbocyanine Cy5-conjugated donkey anti-rabbit antibody (1:200, Jackson Immunoresearch Laboratories). In Figures 4 and 5, the original emission colour of fluorochromes conjugated to secondary antibodies or streptavidin has been sometimes changed to facilitate the observation and analysis of confocal images (ChAT+ always red, other markers green or blue). Images were analyzed with a TCS SP2 AOBS laser scanning confocal microscopy (Leica Microsystems GmbH, Wetzlar). Control sections were processed in the absence of primary antibodies to test the specificity of the secondary antibodies. All of our primary antibodies produced a pattern of immunoreactivity consistent with literature [12,26,46,47].

## Authors' contributions

SC and SL carried out the experiments and analyzed the data. AB designed the experiments and wrote the paper. AA coordinated the work, designed the experiments, analyzed the data and wrote the paper. All authors read and approved the final manuscript.
